# Evaluation of Antepartum Factors for Predicting the Risk of Emergency Cesarean Delivery in Pregnancies Complicated With Placenta Previa

**DOI:** 10.31486/toj.21.0138

**Published:** 2022

**Authors:** Süleyman Cemil Oğlak, Fatma Ölmez, Şeyhmus Tunç

**Affiliations:** ^1^ Department of Obstetrics and Gynecology, Health Sciences University, Gazi Yaşargil Training and Research Hospital, Diyarbakır, Turkey; ^2^ Department of Obstetrics and Gynecology, Health Sciences University, Kanuni Sultan Süleyman Training and Research Hospital, Istanbul, Turkey

**Keywords:** *Cesarean section*, *emergency treatment*, *hemorrhage*, *placenta previa*

## Abstract

**Background:** The optimal delivery timing for patients with placenta previa remains controversial in the literature. To reduce spontaneous vaginal bleeding rates, which occur increasingly with advancing gestational weeks, elective cesarean delivery is advocated between 36^0/7^ and 37^6/7^ weeks of gestation, but this clinical approach does not take into consideration numerous patient variables. Few papers identify the risk factors for emergency cesarean delivery in patients with placenta previa. An enhanced understanding of these variables could help with determining patients at high risk for emergency cesarean delivery and individualizing delivery date scheduling. This study sought to identify predictor variables associated with emergency cesarean delivery in pregnant patients with placenta previa in a tertiary referral hospital. We also investigated differences in maternal and perinatal outcomes between patients with placenta previa who underwent emergency vs planned cesarean delivery.

**Methods:** This retrospective cohort study included 208 singleton pregnancy patients who had a confirmed diagnosis of placenta previa at the time of delivery and who underwent cesarean delivery in our hospital beyond 24 weeks of gestation. To define risk factors of the outcome variable (emergency vs planned cesarean delivery), univariate and multiple logistic regression analysis and adjusted odds ratios with their confidence intervals were calculated.

**Results:** Ninety-seven patients (46.6%) required emergency cesarean delivery, and 111 patients (53.4%) underwent planned cesarean delivery. Antepartum bleeding episode (37.1% and 20.7%, *P*=0.013) and first antepartum bleeding episode ≤28 weeks of gestation (36.1% and 14.4%, *P*<0.001) were significantly higher in the emergency group than the planned group. Antepartum bleeding episode (odds ratio [OR]=1.968, 95% CI 1.001-4.200, *P*=0.042), first antepartum bleeding episode ≤28 weeks of gestation (OR=2.750, 95% CI 1.315-5.748, *P*=0.007), and preoperative hemoglobin level (OR=0.713, 95% CI 0.595-0.854, *P*<0.001) were the independent predictors significantly associated with emergency cesarean delivery.

**Conclusion:** Three factors—antepartum bleeding episode during pregnancy, first antepartum bleeding episode ≤28 weeks of gestation, and lower preoperative hemoglobin level—might be useful in predicting emergency cesarean delivery in pregnancies complicated with placenta previa.

## INTRODUCTION

Placenta previa, the abnormal implantation of placental tissue overlying the endocervical os, is one of the most challenging complications causing maternal and fetal-neonatal morbidity and mortality.^1,2^ The prevalence of placenta previa is estimated to be 5.2 per 1,000 pregnancies.^3^ Pathophysiology remains unknown, but placenta previa appears to be associated with endometrial tissue injury and uterine scarring. This association clarifies the well-known relationship between the growing number of placenta previa cases and the increase in the incidence of cesarean deliveries and assisted reproductive techniques.^4,5^ Various risk factors have also been identified that contribute to the development of placenta previa, including previous placenta previa, multiparity, advanced maternal age, multiple pregnancies, habitual abortion and curettages, and smoking during pregnancy.^6,7^ Most placenta previa cases are detected in the second trimester, and 87% to 95% resolve by the third trimester; complete placenta previa is more likely to persist than incomplete placenta previa.^1,8^

Patients diagnosed with placenta previa clinically manifest with recurrent painless vaginal bleeding before, during, and after delivery, frequently in the third trimester of pregnancy. Maternal hemorrhage is associated with an increased risk of maternal morbidity, including the requirement for additional medications and procedures, blood transfusion, vasa previa, postpartum hemorrhage, sepsis, and longer hospital length of stay (LOS).^9,10^ Concurrently, placenta previa might be complicated by the placental villi invasion beyond the decidua basalis, leading to the placenta accreta spectrum (placenta accreta, increta, or percreta) and increasing the risk of catastrophic hemorrhage, surgical complications, and even death.^11^ In addition to these maternal complications, neonates born to patients with placenta previa have an increased risk of fetal and neonatal complications, principally those associated with preterm delivery and intrauterine growth restriction (IUGR), including lower Apgar scores, low birth weight, and respiratory distress syndrome requiring neonatal intensive care unit (NICU) admission, as well as fetal and neonatal mortality.^12^

The optimal delivery timing for patients with placenta previa remains controversial in the literature. To reduce spontaneous vaginal bleeding rates, which occur increasingly with advancing gestational weeks, elective cesarean delivery is advocated between 36^0/7^ and 37^6/7^ weeks of gestation.^13^ This clinical approach does not take into consideration numerous patient variables, including the history and the severity of the antepartum hemorrhage, prior uterine surgery, prior cesarean delivery, anterior or posterior location of the placenta, the features of the placental edge, and logistical factors.^14^ In a study by Ruiter et al, approximately 40% of patients with placenta previa did not reach the scheduled date for the operation but presented in a preterm or emergency setting and were delivered before the scheduled time.^15^

Few papers identify the risk factors for emergency cesarean delivery in patients with placenta previa.^15-17^ An enhanced understanding of these variables could help us determine patients at high risk for emergency cesarean delivery and individualize delivery date scheduling.

In this study, we sought to identify the predictor variables associated with emergency cesarean delivery in patients with placenta previa. We also investigated differences in maternal and perinatal outcomes between patients with placenta previa who underwent emergency vs planned cesarean delivery.

## METHODS

This retrospective cohort study was conducted at the Diyarbakır Gazi Yaşargil Training and Research Hospital, a tertiary maternal-fetal medicine care center with a level III NICU, between June 2016 and April 2021. The study cohort included 208 singleton pregnancy patients who had a confirmed diagnosis of placenta previa at the time of delivery and who underwent a cesarean delivery in our hospital beyond 24 weeks of gestation. Routinely, all patients with placenta previa are delivered by cesarean section in our hospital after 24 weeks of gestation. In all cases, the antenatal diagnosis of placenta previa was confirmed intraoperatively. We excluded patients with multiple pregnancies and major congenital fetal abnormalities, as well as patients who were referred to our hospital after having delivered elsewhere. We also excluded patients who had emergency cesarean delivery for indications other than the placenta location, including gestational hypertensive disorders, gestational diabetes mellitus, umbilical cord prolapse, and ruptured uterus. The Ethics Committee of the Diyarbakır Gazi Yaşargil Training and Research Hospital approved the study protocol (02.07.2021/820).

According to the Royal College of Obstetricians and Gynaecologists Green-top Guideline No. 27a, the routine second trimester transabdominal scan should include placental localization, thereby identifying patients at risk of placenta previa.^18^ In accordance with the guideline, we generally diagnosed placenta previa during the second trimester scan. All transabdominal ultrasound examinations were performed using a RAB4-8 curvilinear array probe with a frequency range of 4-8 MHz (Voluson P8 Ultrasound System, GE Healthcare Inc.). Following diagnosis, patients were evaluated via transvaginal sonography for placental localization in the third trimester. We confirmed the diagnosis when patients were admitted to the hospital for hemorrhage or delivery and during the operation.

We classified the diagnosis based on the association between the placenta and the internal cervical os: (1) for major placenta previa, the placental tissue overlaid the internal cervical os partially or totally, and (2) for minor placenta previa, the placental tissue was located within 20 mm from the internal cervical os.^19^

According to our clinical protocol, patients with persistent placenta previa in the third trimester were scheduled to undergo a cesarean delivery between 36^0/7^ and 37^6/7^ weeks of gestation. Patients who were delivered at the scheduled time were assigned to the planned group. Patients who were delivered in an emergency setting before the scheduled date were assigned to the emergency group. We categorized the conditions that needed emergency delivery in the following manner: severe antepartum bleeding without labor contractions, premature onset of delivery identified as ruptured membranes or regular labor contractions with or without hemorrhage, and suspected fetal distress defined as nonreassuring fetal heart rate tracing on cardiotocography.^15^ Patients in the emergency group received a single course of betamethasone for lung maturation. The differential diagnosis of placental abruption was principally based on clinical grounds for patients experiencing new-onset antepartum hemorrhage and painful uterine contractions or uterine tenderness, fetal distress or death, or blood clots behind the placenta.^20^

We obtained the following data from the hospital medical records: maternal demographics, obstetric history, pregnancy characteristics, and short-term neonatal and maternal outcomes. Maternal demographics, obstetric history, and pregnancy characteristics included gravidity, parity, previous abortion, previous cesarean delivery, predominant placenta localization (anterior or posterior), type of placenta previa (major or minor), the presence of placenta accreta spectrum, the presence of antepartum bleeding, number of antepartum bleeding episodes, gestational week at hospitalization, and preoperative hemoglobin levels. We recorded maternal outcomes of emergency cesarean delivery, predischarge hemoglobin levels, blood or blood product transfusion requirement, additional surgical (intrauterine sutures, internal iliac artery ligation, B-Lynch suture, and hysterectomy) or nonsurgical procedures (intrauterine balloon tamponade [IUBT] replacement), maternal intensive care unit (ICU) admission, hospital LOS, maternal morbidity, and maternal death. We analyzed neonatal outcomes of gestational week at delivery, birth weight, 1- and 5-minute Apgar scores, small for gestational age (SGA), NICU admission, and neonatal death.

The lower uterine incision was sutured in patients who did not require additional surgical intervention. Intrauterine sutures, IUBT replacement, B-Lynch suture, bilateral internal iliac artery ligation, and/or hysterectomy were conducted in patients requiring additional intervention based on the severity of hemorrhage and uterine atony.

Antepartum bleeding episode was defined as bleeding from the genital tract occurring after 24 weeks of gestation, except for severe bleeding that required emergency cesarean delivery. We defined maternal morbidity as at least 1 of the following complications: postpartum fever (oral temperature >38 °C), wound infection, re-laparotomy, bladder injury, ureter injury, bowel injury, postoperative intra-abdominal infection, and sepsis. We defined SGA as less than the tenth centile for birth weight.^12^ We defined IUGR as estimated fetal weight less than the third centile based on sonographic measurements of fetal biparietal diameter, head circumference, abdominal circumference, and femur length, and end-diastolic flow loss on Doppler examination.^21^

### Statistical Analysis

Statistical analysis was performed using the Statistical Package for Social Sciences, version 22 (IBM). Data are presented as mean ± standard deviation, along with the median and the range (the range of a data set was found by subtracting the minimum value from the maximum value) or as numbers and percentages where appropriate. The homogeneity of variances between the groups was evaluated with the Levene test, and the distribution of continuous variables was evaluated by using Shapiro-Wilk and Kolmogorov-Smirnov tests. Differences between independent groups were analyzed by using the Mann-Whitney U test or *t* test. Chi-square or Fisher exact test was used to compare categorical variables. Before running the regression analysis, correlations between variables were obtained by using Spearman or Pearson correlation coefficient and evaluated with rho and relevant *P* values. To define risk factors for the outcome variable (emergency vs planned cesarean delivery), univariate and multiple logistic regression analysis and adjusted odds ratios (OR) with their confidence intervals were calculated. All covariates with missing data in <20% of observations and a *P* value <0.05 in univariate testing were considered for inclusion in the final multiple regression model and retained if the *P* value was <0.05 or if they demonstrated evidence of significant confounding (>10% change in effect size) and the variables that needed to be adjusted for such as birth weight and birth week. Highly collinear covariates (defined as correlation coefficient >0.6) were not included in the final multivariate model, and these variables are shown in the regression tables. The goodness of fit was assessed by Hosmer-Lemeshow test. A *P* value <0.05 was considered statistically significant for all statistical processes.

## RESULTS

During the study period, 223 patients with placenta previa were delivered in our hospital. Five patients had multiple pregnancies. Three patients had gestational hypertensive disorder. Three patients had intrauterine fetal death. Major fetal abnormality was detected in 2 cases. One patient had placental abruption, and 1 patient experienced uterine rupture. A total of 208 patients with placenta previa completely fulfilled the inclusion criteria for the final evaluation. Of these patients, 97 (46.6%) required emergency cesarean delivery, and 111 (53.4%) underwent planned cesarean delivery. The indications for emergency cesarean section were severe bleeding (84 patients, 86.6%), premature onset of delivery or premature rupture of membranes (10 patients, 10.3%), and nonreassuring fetal heart rate tracing on cardiotocography (3 patients, 3.1%).

### Maternal Characteristics and Outcomes

Demographic and clinical characteristics of the patients are shown in Table 1. We found no significant differences between the 2 groups concerning maternal age, gravidity, parity, number of nulliparous patients, previous abortion, previous cesarean delivery, type of placenta previa, predominant placenta localization, placenta accreta spectrum, and mean number of antepartum bleeding episodes. The occurrence of antepartum bleeding episodes (37.1% and 20.7%, *P*=0.013) and the first antepartum bleeding episode ≤28 weeks of gestation (36.1% and 14.4%, *P*<0.001) were significantly higher in the emergency group than the planned group, respectively.

**Table 1.** Maternal Demographic and Clinical Characteristics

Variable	Emergency Group, n=97	Planned Group, n=111	*P* Value
Maternal age, years, mean ± SD [median, range]	30.48 ± 6.51 [31, 27]	31.38 ± 6.49 [31, 28]	0.319^a^
Gravidity, mean ± SD [median, range]	3.99 ± 2.17 [4, 10]	3.99 ± 2.31 [4, 11]	0.997^a^
Parity, mean ± SD [median, range]	2.34 ± 1.90 [2, 9]	2.62 ± 2.12 [2, 10]	0.319^a^
Nulliparity	13 (13.4)	11 (9.9)	0.432^b^
Previous abortion	38 (39.2)	34 (30.6)	0.196^b^
Number of previous abortions, mean ± SD [median, range]	1.50 ± 0.83 [1, 3]	1.47 ± 0.82 [1, 3]	0.857^b^
Previous cesarean delivery	13 (13.4)	22 (19.8)	0.217^b^
Placenta previa type			0.193^b^
Major	79 (81.4)	82 (73.9)	
Minor	18 (18.6)	29 (26.1)	
Predominant placenta localization			0.236^c^
Anterior	30 (30.9)	41 (36.9)	
Posterior	67 (69.1)	70 (63.1)	
Placenta accreta spectrum	9 (9.3)	4 (3.6)	0.149^b^
Number of antepartum bleeding episodes, mean ± SD [median, range]	1.55 ± 0.69 [1, 2]	1.39 ± 0.65 [1, 2]	0.314^b^
Antepartum bleeding episode, total	36 (37.1)	23 (20.7)	**0.013** ^b^
1	20 (55.6)	16 (69.6)	0.618^c^
2	12 (33.3)	5 (21.7)	
≥3	4 (11.1)	2 (8.7)	
First antepartum bleeding episode ≤28 weeks of gestation	35 (36.1)	16 (14.4)	**<0.001** ^a^

Note: Data are presented as n (%) unless otherwise indicated. Ranges of the mean ± SD data sets were calculated by subtracting the minimum value from the maximum value.

^a^*t* test.

^b^Mann-Whitney U test.

^c^Fisher exact test.

Maternal outcomes are summarized in Table 2. Patients in the emergency group had significantly lower preoperative (10.92 ± 1.95 g/dL) and predischarge (9.71 ± 1.18 g/dL) hemoglobin levels than patients in the planned group (11.86 ± 1.54 g/dL and 10.20 ± 1.25 g/dL, *P*<0.001 and *P*=0.004, respectively). Postoperative blood and blood product transfusion rates were significantly higher in the emergency group vs the planned group. The groups were similar regarding additional surgical procedures and maternal morbidity. One patient in the emergency group required re-laparotomy after cesarean delivery because of excessive bleeding and underwent a hysterectomy. One patient in each group experienced a bladder injury that was repaired intraoperatively. No patient experienced ureter injury, bowel injury, postoperative intra-abdominal infection, or sepsis. No maternal deaths occurred in either group. ICU admission was significantly more common in the emergency group than the planned group (56.7% vs 23.4%, respectively, *P*<0.001). ICU LOS and hospital LOS were significantly longer in the emergency group than the planned group (*P*<0.001).

**Table 2.** Maternal Outcomes

Variable	Emergency Group, n=97	Planned Group, n=111	*P* Value
Preoperative hemoglobin, g/dL, mean ± SD [median, range]	10.92 ± 1.95 [11, 10.70]	11.86 ± 1.54 [11.90, 11]	**<0.001** ^a^
Predischarge hemoglobin, g/dL, mean ± SD [median, range]	9.71 ± 1.18 [9.60, 6.40]	10.20 ± 1.25 [10.10, 6.70]	**0.004** ^b^
Postoperative transfusion, n (%)			
None	54 (55.7)	95 (85.6)	**<0.001** ^c^
Erythrocyte	16 (16.5)	8 (7.2)	
Fresh frozen plasma	1 (1.0)	2 (1.8)	
Erythrocyte + fresh frozen plasma	26 (26.8)	6 (5.4)	
Additional procedures, n (%)			
None	72 (74.2)	97 (87.4)	
Intrauterine sutures	14 (14.4)	8 (7.2)	0.988^c^
B-Lynch	1 (1.0)	0 (0.0)	
Hysterectomy	1 (1.0)	0 (0.0)	
Intrauterine sutures + bladder injury repair	1 (1.0)	1 (0.9)	
Intrauterine sutures + intrauterine balloon tamponade replacement	4 (4.1)	2 (1.8)	
Intrauterine sutures + internal iliac artery ligation	3 (3.1)	2 (1.8)	
Intrauterine sutures + intrauterine balloon tamponade replacement + B-Lynch	1 (1.0)	0 (0.0)	
Intrauterine sutures + uterine lower segment resection	0 (0.0)	1 (0.9)	
Maternal morbidity n (%)			0.200^c^
Postoperative fever	2 (2.1)	3 (2.7)	
Wound infection	0 (0.0)	3 (2.7)	
Re-laparotomy	1 (1.0)	0 (0.0)	
Bladder injury	1 (1.0)	1 (0.9)	
Ureter injury	0 (0.0)	0 (0.0)	
Bowel injury	0 (0.0)	0 (0.0)	
Postoperative intra-abdominal infection	0 (0.0)	0 (0.0)	
Sepsis	0 (0.0)	0 (0.0)	
ICU admission, n (%)	55 (56.7)	26 (23.4)	**<0.001** ^a^
ICU length of stay, days, mean ± SD [median, range]	0.78 ± 0.80 [1, 3]	0.27 ± 0.52 [0, 2]	**<0.001** ^a^
Hospital length of stay, days, mean ± SD [median, range]	2.64 ± 0.85 [3, 7]	2.19 ± 0.53 [2, 3]	**<0.001** ^a^

Note: Ranges of the mean ± SD data sets were calculated by subtracting the minimum value from the maximum value.

^a^Mann-Whitney U test.

^b^*t* test.

^c^Fisher exact test.

ICU, intensive care unit.

### Neonatal Outcomes

Neonatal outcomes of the study cohort are shown in Table 3. Gestational week at delivery, birth weight, and 1- and 5-minute Apgar scores of the infants born to mothers in the emergency group were significantly lower than of the infants born to mothers in the planned group. The neonates in the emergency group had higher NICU admission rates than those in the planned group (36.1% vs 5.4%, *P*<0.001). Eleven neonatal deaths (11.3%) occurred in the emergency group vs zero neonatal deaths in the planned group, and this difference was statistically significant (*P*<0.001). The 11 infants in the emergency group who died were born preterm: 4 were delivered at 23 weeks, 4 at 26 weeks, 1 at 29 weeks, 1 at 30 weeks, and 1 at 32 weeks.

**Table 3.** Neonatal Outcomes

Variable	Emergency Group, n=97	Planned Group, n=111	*P* Value
Gestational week at delivery, mean ± SD [median, range]	33.90 ± 3.98 [35, 18]	37.02 ± 2.22 [38, 4]	**<0.001** ^a^
Birth weight, g, mean ± SD [median, range]	2,310.41 ± 799.13 [2,450, 3,400]	2,930.81 ± 538.41 [3,000, 770]	**<0.001** ^b^
Apgar score, 1 min, mean ± SD [median, range]	6.97 ± 2.36 [8, 9]	8.00 ± 1.33 [8, 6]	**<0.001** ^a^
Apgar score, 1 min, <7, n (%)	21 (21.6)	6 (5.4)	**0.001** ^a^
Apgar score, 5 min, mean ± SD [median, range]	8.14 ± 2.32 [9, 10]	9.10 ± 1.35 [9, 4]	**<0.001** ^a^
Apgar score, 5 min, <7, n (%)	13 (13.4)	1 (0.9)	**<0.001** ^a^
Small for gestational age, n (%)	7 (7.2)	5 (4.5)	0.403^a^
NICU admission, n (%)	35 (36.1)	6 (5.4)	**<0.001** ^a^
Neonatal death, n (%)	11 (11.3)	0 (0)	**<0.001** ^a^

Note: Ranges of the mean ± SD data sets were calculated by subtracting the minimum value from the maximum value.

^a^Mann-Whitney U test.

^b^*t* test.

NICU, neonatal intensive care unit.

### Regression Analysis Results

We performed univariate and multiple logistic regression analysis to adjust the groups for gestational week at delivery and birth weight (Table 4). Following the multiple logistic regression analysis, 1-minute Apgar score (OR 1.345, 95% CI 0.813-2.173, *P*=0.227), number of neonates with a 1-minute Apgar score <7 (OR 0.842, 95% CI 0.091-7.764, *P*=0.879), 5-minute Apgar score (OR 0.958, 95% CI 0.698-1.316, *P*=0.793), number of neonates with a 5-minute Apgar score <7 (OR 3.383, 95% CI 0.222-5.473, *P*=0.380), and neonatal death (OR 1.843, 95% CI 0.127-26.653, *P*=0.654) were not significantly different between the groups.

**Table 4.** Univariate and Multiple Logistic Regression Results for Neonatal Outcomes After Adjusting for Gestational Week at Delivery and Birth Weight

Variable	Univariate Logistic Regression Odds Ratio (95% CI)	*P* Value	Multiple Logistic Regression Odds Ratio (95% CI)	*P* Value
Gestational week at delivery	0.661 (0.568-0.770)	**<0.001**	0.713 (0.570-0.891)	**0.003**
Birth weight	0.999 (0.998-0.999)	**<0.001**	1.000 (0.999-1.001)	0.513
Apgar score, 1 min	0.709 (0.578-0.870)	**0.001**	1.345 (0.813-2.173)	0.227
Apgar score, 1 min, <7	4.836 (1.862-12.555)	**<0.001**	0.842 (0.091-7.764)	0.879
Apgar score, 5 min	0.699 (0.555-0.881)	**0.002**	0.958 (0.698-1.316)	0.793
Apgar score, 5 min, <7	17.229 (2.210-34.244)	**<0.001**	3.383 (0.222-5.473)	0.380
NICU admission	9.879 (3.932-24.818)	**<0.001**	3.252 (1.003-10.547)	**0.043**
Neonatal death	14.070 (1.782-111.107)	**0.012**	1.843 (0.127-26.653)	0.654

NICU, neonatal intensive care unit.

However, in multiple logistic regression analysis, NICU admission rates (OR 3.252, 95% CI 1.003-10.547, *P*=0.043) were found to be significantly associated with emergency cesarean delivery after adjusting for the gestational week at delivery and birth weight.

We conducted univariate analysis and multiple logistic regression analysis by adjusting the groups for gestational week at delivery and birth weight to clarify factors independently associated with emergency cesarean delivery in patients with placenta previa (Table 5). According to our multiple logistic regression analysis, antepartum bleeding episode (OR 1.968, 95% CI 1.001-4.200, *P*=0.042), first antepartum bleeding episode ≤28 weeks of gestation (OR 2.750, 95% CI 1.315-5.748, *P*=0.007), and preoperative hemoglobin level (OR 0.713, 95% CI 0.595-0.854, *P*<0.001) were the predictors significantly associated with emergency cesarean delivery.

**Table 5.** Univariate and Multiple Logistic Regression Analysis for Predicting Emergency Cesarean Delivery After Adjusting for Gestational Week at Delivery and Birth Weight

Variable	Univariate Logistic Regression Odds Ratio (95% CI)	*P* Value	Multiple Logistic Regression Odds Ratio (95% CI)	*P* Value
Antepartum bleeding episode	2.558 (1.219-4.184)	**0.010**	1.968 (1.001-4.200)	**0.042**
First antepartum bleeding episode ≤28 weeks of gestation	3.552 (1.711-6.567)	**<0.001**	2.750 (1.315-5.748)	**0.007**
Preoperative hemoglobin	0.725 (0.607-0.865)	**0.001**	0.713 (0.595-0.854)	**<0.001**

## DISCUSSION

In this examination of the variables that might be related to emergency delivery in pregnancies complicated with confirmed placenta previa, we detected 3 antepartum variables significantly associated with emergency delivery: antepartum bleeding episode during pregnancy, first antepartum bleeding episode ≤28 weeks of gestation, and preoperative hemoglobin level. In the comparison of maternal and neonatal outcomes of emergency and planned cesarean delivery, we demonstrated that the planned group had significantly better maternal outcomes (including higher preoperative and predischarge hemoglobin levels, the requirement for postoperative transfusion, lower ICU admission rates, shorter ICU LOS and hospital LOS) and better neonatal outcomes (including higher birth weight, higher Apgar scores, lower NICU admission rates, and a lower neonatal death rate) than the emergency group.

Recommendations regarding the optimum timing of delivery have not been established in the literature.^22^ When planning the delivery time for these patients, neonatal and maternal adverse outcomes must be balanced with the risks of continuing the pregnancy.^23^ Durukan et al stated that delivery timing for patients with placenta previa in tertiary centers might be delayed if timing is individualized based on the maternal status.^24^ In their clinic, patients with placenta previa are usually scheduled to undergo cesarean delivery after 38 weeks out of concern for fetal lung maturation, and maternal mortality is low. However, scheduling delivery in the later weeks of gestation has the possibility of failure; patients may not reach the planned delivery date and require emergent admission.^24^ Balayla et al concluded that patients with placenta previa delivered at the early-term period between 37 and 38^6/7^ weeks of gestation had significantly fewer neonatal complications and no greater risk than the placenta previa patients delivered at the late-preterm period, namely 35 to 36^6/7^ weeks of gestation.^22^ In 2019, the American College of Obstetricians and Gynecologists (ACOG) suggested scheduling delivery for patients with placenta previa at 36^0/7^ to 37^6/7^ weeks of gestation.^23^ In cases of suspected placenta accreta spectrum, ACOG recommended delivery at 34^0/7^ to 35^6/7^ weeks of gestation. In accordance with these recommendations, in our clinic, we generally schedule patients with placenta previa for cesarean delivery between 36^0/7^ to 37^6/7^ weeks of gestation based on an individualized approach.

Precisely predicting whether a patient with placenta previa will present with bleeding in the emergency room is not possible, nor can we predict the gestational week, the amount of blood, or the recurrence of bleeding episodes.^22^ Gedik Özköse et al observed that 33.2% of patients with placenta previa underwent unscheduled delivery in the preterm period principally because of vaginal bleeding, labor pains, and fetal distress.^25^ Erfani et al showed that 42.8% of pregnant patients with placenta previa (without placenta accreta spectrum) required emergent delivery because of premature labor contractions or vaginal bleeding.^26^ Ruiter et al showed that of all patients with placenta previa who were planned for cesarean delivery after 37 weeks of gestation, 43% were unable to reach the scheduled delivery date, and the reason for 55% of these patients was severe hemorrhage.^15^ Love et al demonstrated that patients with antepartum hemorrhage were significantly more likely to require emergency delivery, but major placenta previa and the number of bleeding episodes did not predict emergency delivery.^27^ Fishman et al concluded that antepartum bleeding before 34 weeks of gestation is a strong predictor of preterm birth and emergency delivery.^16^ In the study by Luangruangrong et al, patients with placenta previa complicated by antepartum hemorrhage had significantly higher risks of preterm birth, emergency delivery, and blood transfusion.^28^ Pivano et al reported that major placenta previa, ≥3 antepartum bleeding episodes, first bleeding before 29 weeks of gestation, and moderate or severe antepartum bleeding episodes were significantly associated with emergency delivery.^17^ Ruiter et al identified a history of cesarean delivery, the number of antepartum bleeding episodes, and the requirement for blood transfusion as independent predictors for an emergency delivery.^15^ In our study, 46.6% of patients with placenta previa failed to reach the scheduled delivery time and were delivered preterm because of severe bleeding, nonreassuring fetal heart rate status, early onset of uterine contractions, or preterm premature rupture of membranes. Antepartum bleeding episode, first antepartum bleeding episode ≤28 weeks of gestation, and lower preoperative hemoglobin level were the significant risk factors for emergent deliveries in our study cohort. We were unable to calculate the estimated or total blood loss in our patients because of the retrospective design of the study. Because of interobserver differences, the subjective visual estimation of the severity of bleeding episodes by clinicians is associated with a 30% to 50% underestimation of blood loss.^29^

Grönvall et al observed that preterm delivery was more common and blood loss was heavier among women with major placenta previa.^19^ However, as in our study, Ruiter et al and Tuzovic detected no difference in preterm delivery rates regarding the type of placenta previa.^15,30^ We suggest that variations in outcomes regarding the placenta previa type could be attributable to the diverse categorization of placenta previa types and a wide variation in clinical management.

In the Erfani et al study, planned and emergent cesarean deliveries for patients with placenta previa but without placenta accreta spectrum had similar maternal outcomes, including blood transfusion, additional surgical intervention, and composite maternal morbidity.^26^ However, Durukan et al stated that preoperative and predischarge hemoglobin levels were significantly lower, while blood transfusions, additional intraoperative interventions, and ICU admission were significantly higher in the emergency group vs the planned group.^24^ Likewise, in our population, preoperative and predischarge hemoglobin levels were significantly lower, blood transfusion and ICU admission were significantly higher, and ICU LOS and hospital LOS were significantly longer in the emergency group vs the planned group. As would be expected, patients in the emergency group were delivered earlier than the planned group and had significantly more adverse neonatal outcomes, including lower birth weight, lower Apgar scores, higher NICU admission rates, and higher frequency of neonatal death. NICU admission rate was not associated with birth weight or birth week because when we adjusted for these variables, the emergency group still had a higher rate of NICU admission than the planned group. This difference might be attributable to maternal status. However, Lal and Hibbard concluded that increased neonatal morbidity and NICU admission are related to the gestational age and birth weight of the newborn rather than the maternal status of placenta previa.^12^

Limitations and strengths of the current study need to be acknowledged. The principal limitation of this study is its retrospective design. Also, because our hospital is a tertiary referral center, a preadmission selection bias may exist. The major strength of this study is that the placenta previa diagnosis was confirmed for all patients at the time of delivery, in contrast to studies that solely used the results of the most recent antenatal ultrasound. Second trimester placenta previa resolves during the third trimester in 87% to 95% of cases; we can confirm that patients with resolved placenta previa were not included in the study population.

## CONCLUSION

Antepartum factors, including a bleeding episode during pregnancy, a first bleeding episode ≤28 weeks of gestation, and lower preoperative hemoglobin level might be useful in predicting emergency cesarean delivery in pregnancies complicated with placenta previa.

## Notes

*This article meets the Accreditation Council for Graduate Medical Education and the American Board of Medical Specialties Maintenance of Certification competencies for Patient Care, Medical Knowledge, and Practice-Based Learning and Improvement.*
